# Long Noncoding RNA FAM225A Promotes Esophageal Squamous Cell Carcinoma Development and Progression via Sponging MicroRNA-197-5p and Upregulating NONO

**DOI:** 10.7150/jca.51292

**Published:** 2021-01-01

**Authors:** Pengyuan Zhu, Haitao Huang, Shaorui Gu, Zhenchuan Liu, Xin Zhang, Kaiqin Wu, Tiancheng Lu, Lei Li, Chenglai Dong, Chongjun Zhong, Yongxin Zhou

**Affiliations:** 1 Department of thoracic and Cardiovascular Surgery, the Second Affiliated Hospital of Nantong University, School of Medicine, Nantong University, Nantong, 226001, China.; 2 Department of thoracic and Cardiovascular Surgery, Tongji Hospital, Tongji University School of Medicine, Shanghai, 200065, China.

**Keywords:** FAM225A, microRNA-197-5p, NONO, ESCC

## Abstract

Esophageal squamous cell carcinoma (ESCC) is the major subclass of esophageal cancer and one of the most life-threatening malignancies with high morbidity and mortality. Long noncoding RNAs (lncRNAs) participate in tumorigenesis and metastasis of various tumors. Here, we investigated the function of a newly identified lncRNA FAM225A in ESCC. LncRNA FAM225A expression was significantly higher in ESCC and predicted poor prognosis of ESCC patients. We confirmed that upregulation of FAM225A in ESCC and overexpression of FAM225A was associated with poor outcome in ESCC patients using TCGA ESCC cohort. Knockdown of FAM225A significantly inhibited cell growth, migration and invasion of ESCC cells in vitro and inhibited ESCC xenograft development in vivo. Mechanistically, we demonstrated that lncRNA FAM225A functioned as a competing endogenous RNA (ceRNA) via sponging miR-197-5p. LncRNA FAM225A exerted its regulatory function on ESCC proliferation and metastasis via modulating expression of miR-197-5p. MiR-197-5p overexpression antagonized the function of FAM225A, with decreased cell growth and invasion. Moreover, we identified that RNA binding protein NONO was a direct target of miR-197-5p and miR-197-5p negatively regulated NONO expression and TGF-β signaling in ESCC cells. In summary, our findings suggest that lncRNA FAM225A promotes ESCC development and progression via sponging miR-197-5p and upregulating NONO expression. These results suggest that lncRNA FAM225A could be explored as a new therapy target in ESCC treatment.

## Introduction

Esophageal squamous cell carcinoma (ESCC) is the major subclass of esophageal cancer and one of the most life-threatening malignancies with high morbidity and mortality 1, 2. In the past decade, the prognosis of ESCC patients have been improved significantly due to the advances in diagnosis technology and therapies such as chemotherapy, radiotherapy and molecular targeted therapy 3, 4. However, the overall 5-year survival rates are still below 20% in ESCC recurrent patients or ESCC patients with metastasis 5. Hence, it is crucial to understand the molecular mechanisms of ESCC tumorigenesis and progression, and develop novel therapeutic treatment for ESCC.

Long noncoding RNAs (lncRNAs) are a group of long transcripts (> 200 nt) without protein translation 6. Accumulating evidence has shown that lncRNAs have important regulatory functions in tumorigenesis and metastasis 7, 8. Dysregulated expression of lncRNAs has been reported in ESCC 9, 10. LncRNAs function as tumor suppressors or oncogenes in the tumorigenesis and metastasis of ESCC 9, 11, 12. For example, lncRNA HOTTIP was highly expressed in ESCC and promotes metastasis of ESCC via activating of epithelial-mesenchymal transition (EMT) 13. CBP/P300 was reported to enhance the expression of lncRNA linc00460 and promote ESCC carcinogenesis 14. In the contrast, lncRNA RP11-766N7.4 acts as a tumor suppressor and suppresses EMT process in ESCC 15. Sun et al. demonstrated that lncRNA CASC2 inhibited ESCC development and progression via increasing the expression of SOCS1 16.

LncRNA FAM225A is a newly identified lncRNA involved in multiple cancer development and progression 17, 18. In nasopharyngeal carcinoma, lncRNA FAM225A functions as a ceRNA to sponge miR-590-3p and miR-1275 expression, and subsequently upregulates ITGB3 expression to promote tumor development and metastasis 17. FAM225A could also regulate NOTCH3 expression and sponge miR-613 expression to enhance the colorectal cancer development 18. Nevertheless, the expression pattern and function of lncRNA FAM225A in ESCC has not been studied yet.

In this study, we revealed that lncRNA FAM225A expression was significantly upregulated in ESCC tissues and cell lines and high expression of FAM225A predicted poor prognosis of ESCC patients. Silencing FAM225A suppressed ESCC cell growth and invasive phenotype and inhibited ESCC xenograft development. Moreover, we demonstrated that FAM225A exerted its function via sponging miR-197-5p expression and upregulating the expression of RNA binding protein NONO. Our results suggest that FAM225A/miR-197-5p/NONO axis plays a critical role in ESCC development and progression, which might serve as a promising therapy target for ESCC treatment.

## Materials and Methods

### Patient specimens

ESCC tissues and adjacent normal tissues (118 matched-pairs) were collected from ESCC patients at The Second Affiliated Hospital of Nantong University from 2004 year to 2018 year (NTU cohort). The fresh collected tissues were snap-frozen in liquid nitrogen until further use. All patients provided the written informed consent and the study was reviewed and approved by the Institutional Ethical Review Board of The Second Affiliated Hospital of Nantong University.

### Cell culture

Four ESCC cell lines (EC9706, KYSE30, KYSE450, and KYSE510) and control cell Het-1A were from Cell Bank, China Academy of Sciences (Shanghai, China). Cells were cultured in DMEM medium supplemented with 10% fetal bovine serum (Invitrogen, USA) and 1% antibiotics (Thermo Scientific, USA) at 37 °C in a 5% CO_2_ cell incubator.

### Transfection

The shRNA targeting FAM225A and negative control were purchased from GenePharma company (Shanghai, China). MiR-197-5p mimics, miR-197-5p inhibitor and relative negative control were obtained from RiboBio company (Guangzhou, China). Overexpression plasmids (pcDNA3.1-FAM225A and pcDNA3.1-NONO) and empty vector pcDNA3.1 were purchased from GeneCopoeia company (Guangzhou, China). Transfection was done using Fugene HD reagent (Roche, Switzerland) following the manufacturer's protocol.

### Quantitative real-time PCR (qRT-PCR)

RNA was isolated from tissues samples or cultured cells using Trizol (Thermo Scientific, USA) and reverse-transcribed using Super Transcriptase III (Invitrogen, USA). PowerUP SYBR master mix (Applied Biosystems, USA) was used for qPCR analysis. The relative expression was analyzed by 2^-ΔΔCT^ methods. The sequences of primers were listed below: FAM225A, 5'-AAGTCCAGCTGATGCCAGAC-3' and 5'-CACAAGCTTCATCCGAGGGT-3'; miR-197-5p, 5'-CAGTGCAGGGTCCGAGGT-3' and 5'-AACAAGCGGGTAGAGAGGGC-3'; NONO, 5'-CTGATGCGAGAGAGCAGGAG-3' and 5'-ATCCGGCATCATAGTGGCAG-3'; GAPDH, 5'-GTCAAGGCTGAGAACGGGAA-3' and 5'-AAATGAGCCCCAGCCTTCTC-3'.

### Western blot

Protein lysate preparation and western blot analysis were performed as described previously 19. The following primary antibodies were used in the study: NONO (Abcam, ab266244, USA), E-cadherin (Abcam, ab215715, USA), GAPDH (Abcam, ab181602, USA).

### Luciferase reporter assay

Wild type (WT) or mutated (MUT) 3'-UTR of FAM225A or NONO was amplified and constructed into psiCHECK2 luciferase vector (Promega, USA). KYSE510 cells were transfected with luciferase vector, together with miR-197-5p mimics. The relative luciferase activity was analyzed 48 hours post transfection using a Dual-luciferase reporter assay kit (Promega, USA).

### Cell growth assay

Cell growth was analyzed by CCK-8 assay using cell counting kit-8 (Sigma-Aldrich, USA) and colony formation assay as previously described 19.

### Wound-healing assay

Transfected cells were cultured in 6-well plates until fluent. The artificial wound was created with a sterile tip and floating cells were washed away with PBS. Cells were cultured for another 48 hours in serum-free medium and cell migration was photographed and calculated at 0 h and 48 h.

### Transwell assay

Transfected cells (1 × 10^5^) were suspended in 200 μL serum free medium and added to the top layer of a transwell chamber pre-coated with Matrigel (Corning, USA). Bottom chamber was filled with 500 μL complete medium with 10% FBS. The invaded cells were fixed, stained and counted under a light microscope after 24 h culture.

### Immunohistochemical staining

Tissue sections from different groups were deparaffinized, rehydrated and blocked with 1% FBS. Then, tissue sections were stained with anti-Ki-67 antibodies (Abcam, ab270650) overnight at 4°C. The staining was visualized using the DAKO EnVision System (DAKO, Denmark).

### Xenograft tumor model

All animal experiments were reviewed and approved by the Institutional Animal Care and Use Committee of The Second Affiliated Hospital of Nantong University. Xenograft tumor model was established by inoculating KYSE510 cells (5 × 10^6^) stably transfected with sh-FAM225A or negative control into nude mice (male, 5 weeks old). Tumor growth was measured every week. On week 5, mice were euthanized and xenograft tumors were dissected and weighted. The tumor tissue sections were fixed and paraffin-embedded for hematoxylin and eosin (H&E) and Ki-67 staining analysis.

### In situ hybridization

In Situ Hybridization (ISH) was conducted using an In situ hybridization kit (Boster, China). Tissue sections were processed to de-paraffinization, dehydration and digested with pepsin. Subsequently, sections were incubated with FAM225A-specific probe at 50°C overnight. Sections were further incubated with biotinylated anti-Digoxin (Boster, China), stained with biotinylated peroxidase, and visualized with 3,3'-diaminobenzidine substrate.

### Statistical analysis

The results of at least three experiments were shown as mean ± standard deviation (SD). Kaplan-Meier analysis was performed to analyze the overall survival (OS) and disease-free survival (DFS) of ESCC patients with high or low FAM225A expression. The statistical analysis was conducted using GraphPad Prism (V6, Prism, USA) and a P value < 0.05 was considered statistical significance.

## Results

### LncRNA FAM225A is highly expressed in ESCC and predicts poor prognosis

**Figure 1:**
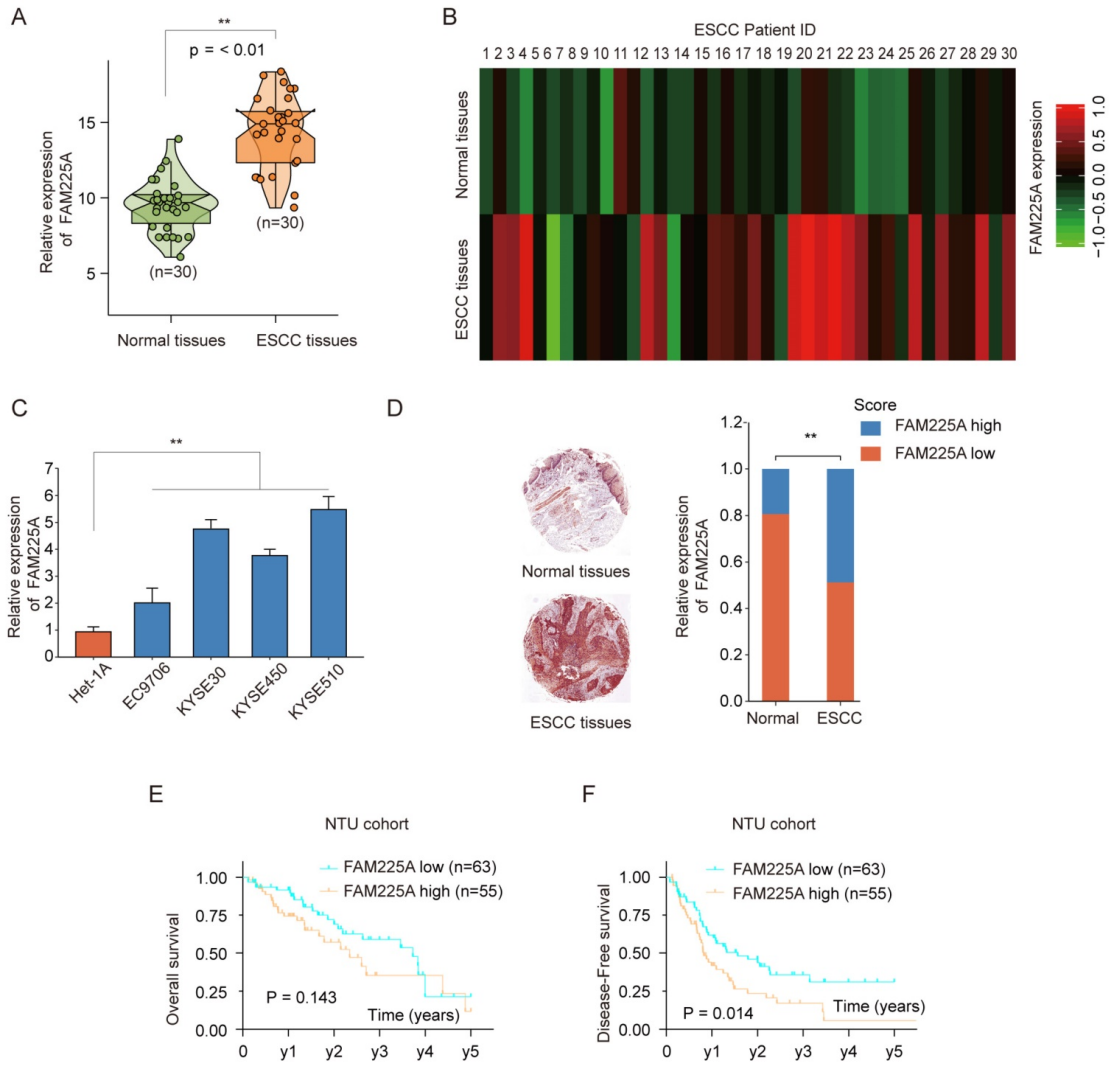
** LncRNA FAM225A is upregulated in ESCC and predicts poor prognosis.** (A) The expression of lncRNA FAM225A in 30-paired ESCC tissues and adjacent normal tissues was analyzed by qRT-PCR. (B) Heat map illustrated the microarray analysis of relative expression of lncRNA FAM225A in ESCC tissues and adjacent normal tissues from 30 ESCC patients. (C) The expression of lncRNA FAM225A in ESCC cell lines (EC9706, KYSE30, KYSE450, and KYSE510) and control cell line Het-1A was examined by qRT-PCR. (D) In situ hybridization (ISH) was performed to examine the expression of lncRNA FAM225A in ESCC tissues and the relative expression of lncRNA FAM225A was scored as 1 to 5 based on the staining intensity. Relative ISH staining of lncRNA FAM225A in normal tissues and ESCC tissues and the distribution of different scores of FAM225A ISH staining intensity were shown. (E) Kaplan-Meier analysis of overall survival (OS) in ESCC patients with high or low lncRNA FAM225A expression was performed. (F) Kaplan-Meier analysis of Disease-free survival (DFS) in ESCC patients with high or low lncRNA FAM225A expression was performed. ** P < 0.01.

To investigate the lncRNA FAM225A expression in ESCC, we performed qPCR analysis in 30-paired ESCC tissues and adjacent tissues. LncRNA FAM225A expression was much higher in ESCC tissues than that in normal tissues (**Figure 1A**). Microarray analysis using 30-paired ESCC tissues and adjacent tissues also confirmed high expression of FAM225A in ESCC tissues (**Figure 1B**). In addition, lncRNA FAM225A expression was markedly higher in ESCC cell lines (EC9706, KYSE30, KYSE450, and KYSE510) than in control Het-1A cells (**Figure 1C**). In situ hybridization (ISH) was performed using ESCC tissue microarray and the relative expression of lncRNA FAM225A was scored as 1 to 5 according to the staining intensity. The ISH results suggested that ESCC tissues had significantly upregulated lncRNA FAM225A expression than that in adjacent tissues (**Figure 1D**). Furthermore, Kaplan-Meier analysis showed that ESCC patients with higher lncRNA FAM225A expression had no statistic difference for overall survival (OS) (**Figure 1E**), while there was significant worse disease-free survival (DFS) in comparison with patients had low lncRNA FAM225A expression (**Figure 1F**).

**Figure 2:**
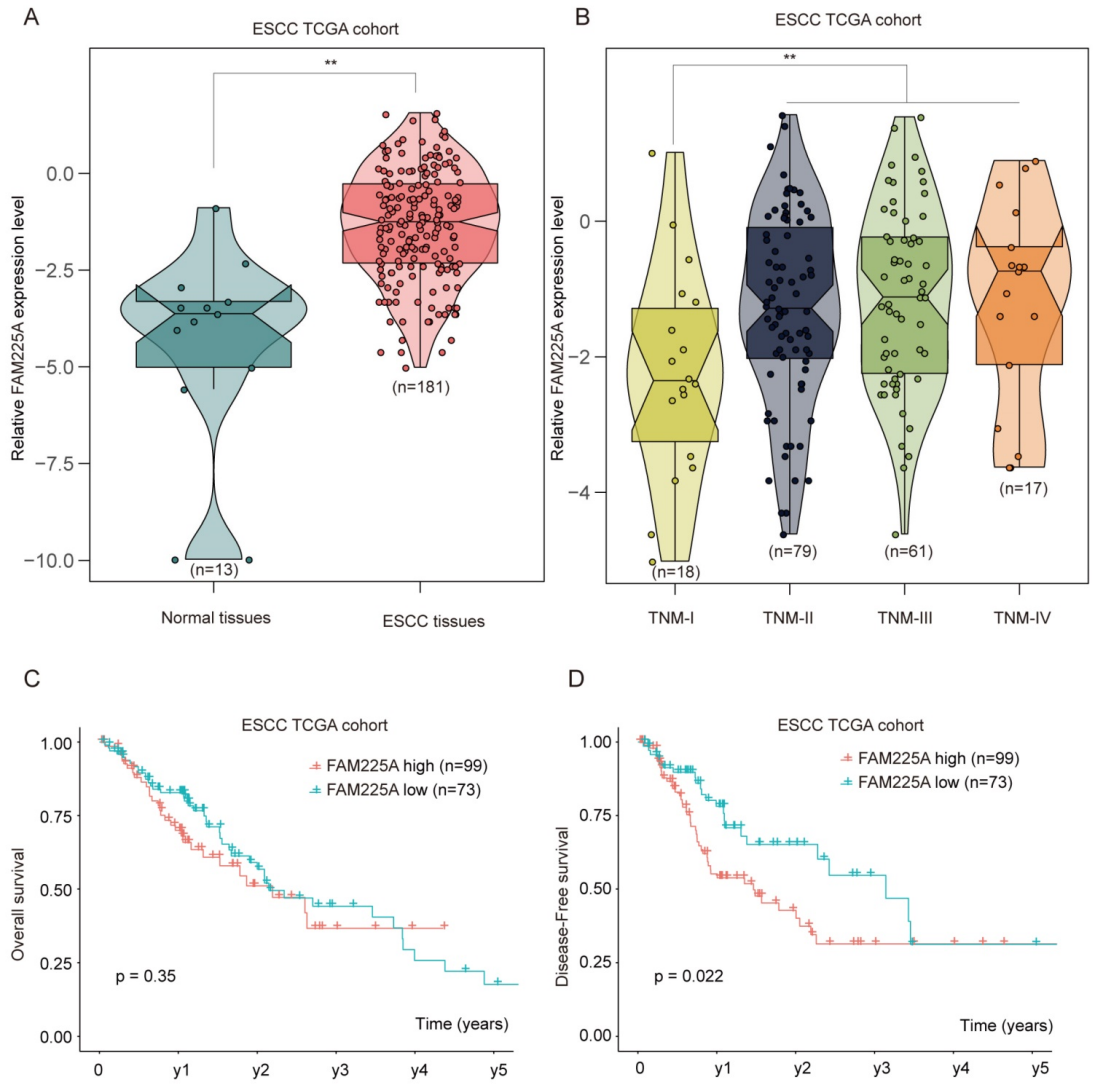
** ESCC TCGA datasets analysis indicates high expression of lncRNA FAM225A correlates poor outcome in ESCC patients.** (A) RNA sequencing data of ESCC from the Cancer Genome Atlas (TCGA) was analyzed and the relative expression of lncRNA FAM225A in normal tissues and ESCC tissues was shown. (B) The relative expression of lncRNA FAM225A in ESCC tissues with different TNM stages was analyzed in ESCC TCGA cohort. (C) Kaplan-Meier analysis of OS in ESCC patients with high or low lncRNA FAM225A expression in ESCC TCGA cohort was performed. (D) Kaplan-Meier analysis of DFS in ESCC patients with high or low lncRNA FAM225A expression in ESCC TCGA cohort was performed. ** P < 0.01.

To further validate the expression of lncRNA FAM225A in ESCC, RNA sequencing data of ESCC from the Cancer Genome Atlas (TCGA) was analyzed and ESCC tissues had significantly higher lncRNA FAM225A expression (**Figure 2A**). Intriguingly, we found that ESCC tissues with advanced TNM stages had higher lncRNA FAM225A expression levels than those in ESCC tissues of TNM stage I (**Figure 2B**). Consistently, ESCC patients with high lncRNA FAM225A level had worse DFS, but not OS, compared with patients with low lncRNA FAM225A levels in ESCC TCGA cohort (**Figure 2C and D**). Our results suggested that lncRNA FAM225A expression was overexpressed in ESCC tissues and predicted unfavorable prognosis of ESCC patients.

### Knockdown of lncRNA FAM225A suppresses ESCC cell proliferation, migration and invasion

**Figure 3:**
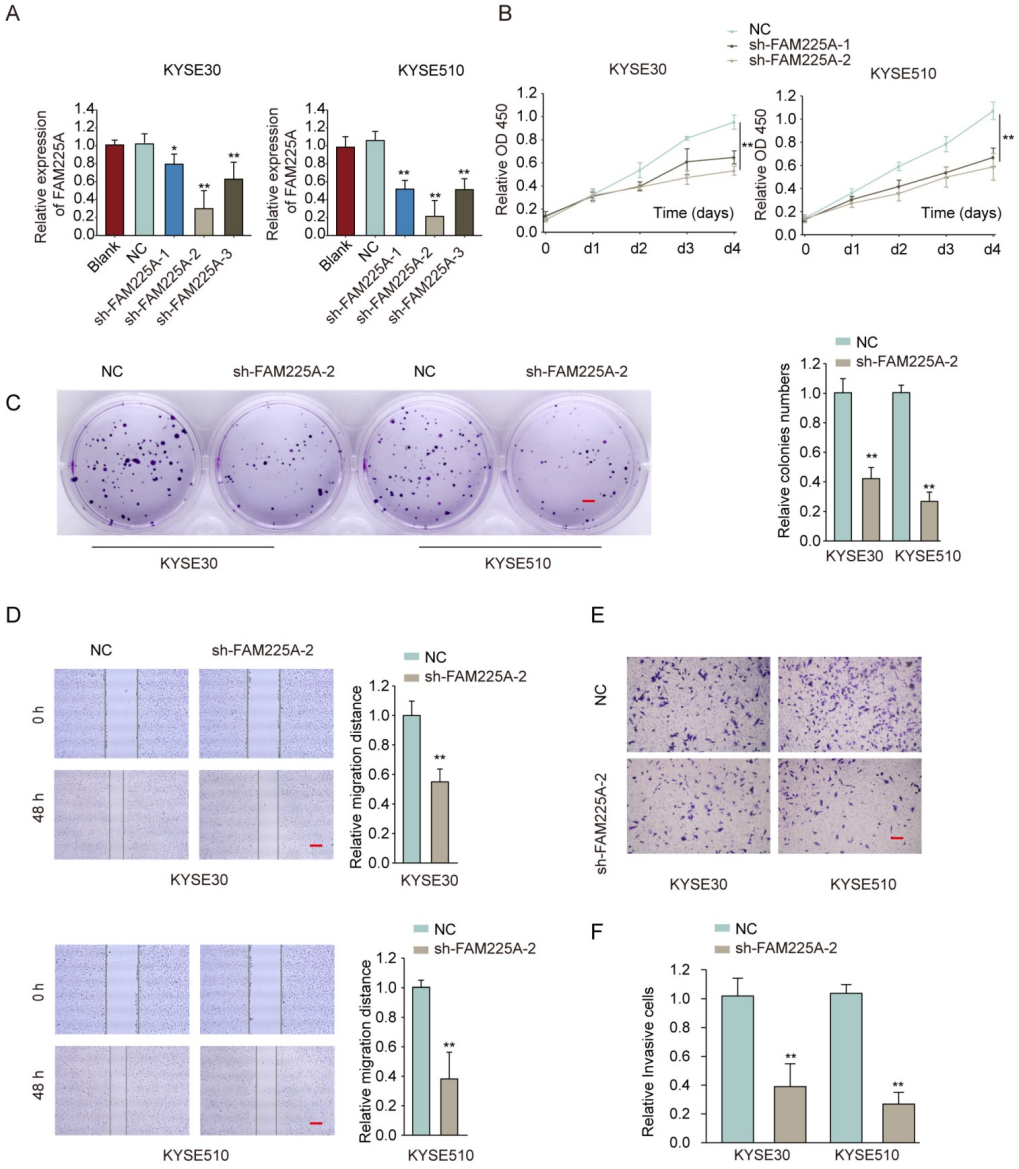
** Knockdown of lncRNA FAM225A suppresses ESCC cell proliferation, migration and invasion.** (A) KYSE30 or KYSE510 cells were transfected with negative control (NC), sh-FAM225A-1/2/3, or left untreated (blank). The relative expression of FAM225A was examined by qRT-PCR 48 hours later. (B-F) KYSE30 or KYSE510 cells were transfected with NC, sh-FAM225A-1, or sh-FAM225A-2. (B) Cell proliferation was analyzed by CCK-8 assay at indicated time. (C) Cell growth was analyzed by colony formation. (D) Cell migration was analyzed by wound-healing assay. (E) Cell invasion was analyzed by transwell assay. * P < 0.05, ** P < 0.01.

To study the function of lncRNA FAM225A on ESCC cell proliferation and metastasis ability, two ESCC cell lines (KYSE30 and KYSE510) with highest levels of lncRNA FAM225A, were transfected with shRNA targeting lncRNA FAM225A to knockdown its expression (**Figure 3A**). Functional assays showed that knockdown of lncRNA FAM225A significantly dampened cell proliferation and colony formation of ESCC cells (**Figure 3B and 3C**). Furthermore, wound-healing assay and transwell assay demonstrated that suppression of FAM225A dampened the migration and invasion capabilities of ESCC cells (**Figure 3D-3F**). These findings indicated that lncRNA FAM225A functioned as an oncogene and promoted ESCC cell proliferation, migration and invasion.

### Knockdown of lncRNA FAM225A inhibits ESCC tumorigenesis in vivo

**Figure 4:**
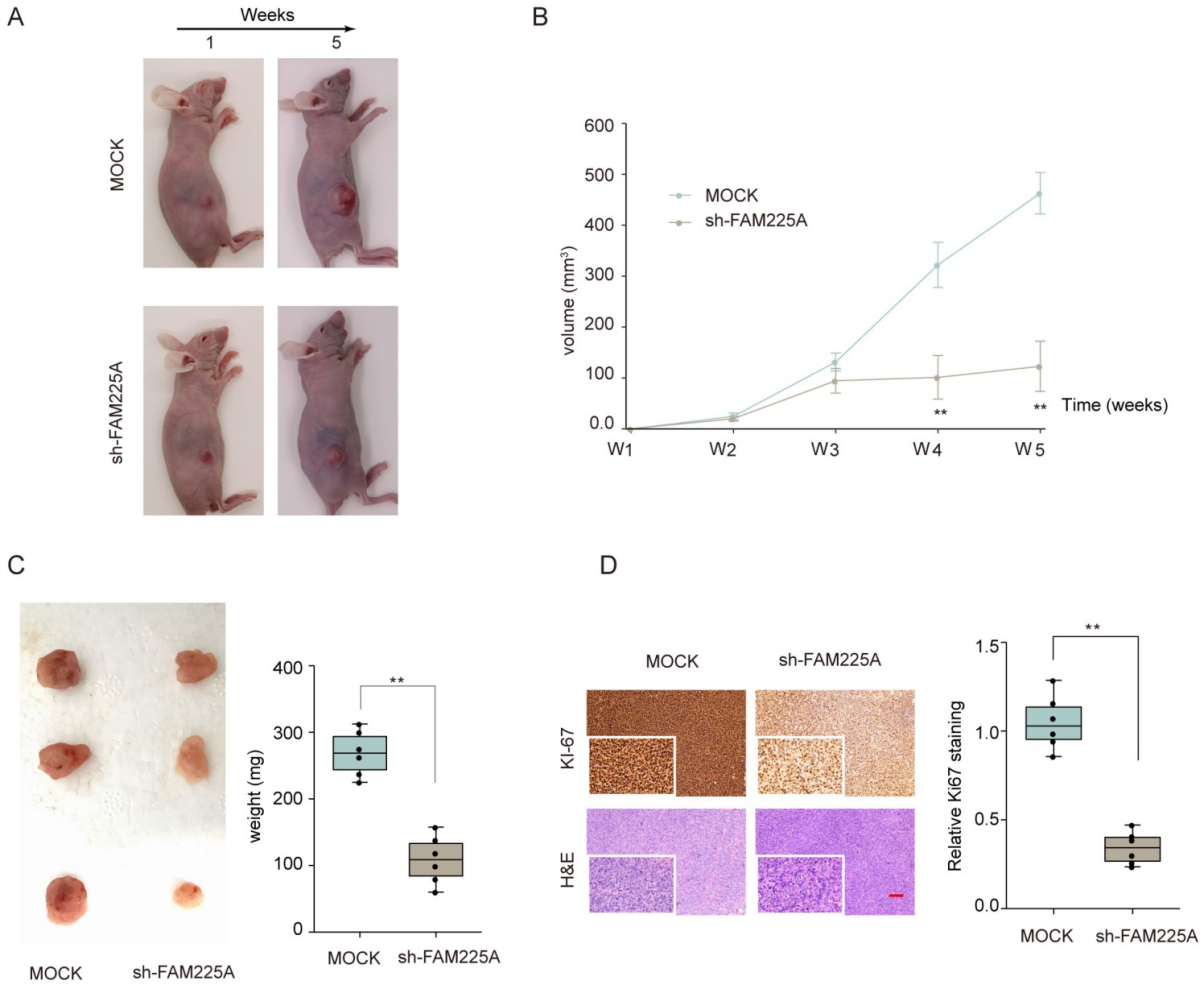
** Knockdown of lncRNA FAM225A inhibits ESCC xenograft tumor development in vivo.** KYSE510 cells stably transfected with sh-FAM225A or mock control were transplanted into the right flank of nude mice to establish ESCC xenograft tumor model. (A) The representative images of nude mice in different groups at week 1 and weeks 5 were shown. (B) Tumor growth was monitored at indicated time points. (C) Tumor weights from different groups were analyzed. (D) The representative immunohistochemical staining of Ki-67 and H&E staining of xenograft tumor sections and the quantification of Ki-67 expression were analyzed. ** P < 0.01.

We also evaluated the function of lncRNA FAM225A on ESCC tumorigenesis in vivo. Xenograft tumor model of ESCC was established by transplanting stable transfected KYSE510 cells (Mock or sh-FAM225A) into nude mice. As shown in **Figure 4A-4C**, knockdown of lncRNA FAM225A significantly inhibited ESCC tumor growth, with markedly delayed tumor development and decreased tumor weights in sh-FAM225A group. In addition, immunohistochemical staining suggested that knockdown of FAM225A markedly decreased the expression of proliferation marker Ki-67 in xenograft tumors. H&E counter-staining was performed to visualize the tumor tissues (**Figure 4D**). Our data indicated that knockdown of FAM225A suppressed ESCC tumorigenesis in vivo.

### LncRNA FAM225A functions as a ceRNA via sponging miR-197-5p

**Figure 5:**
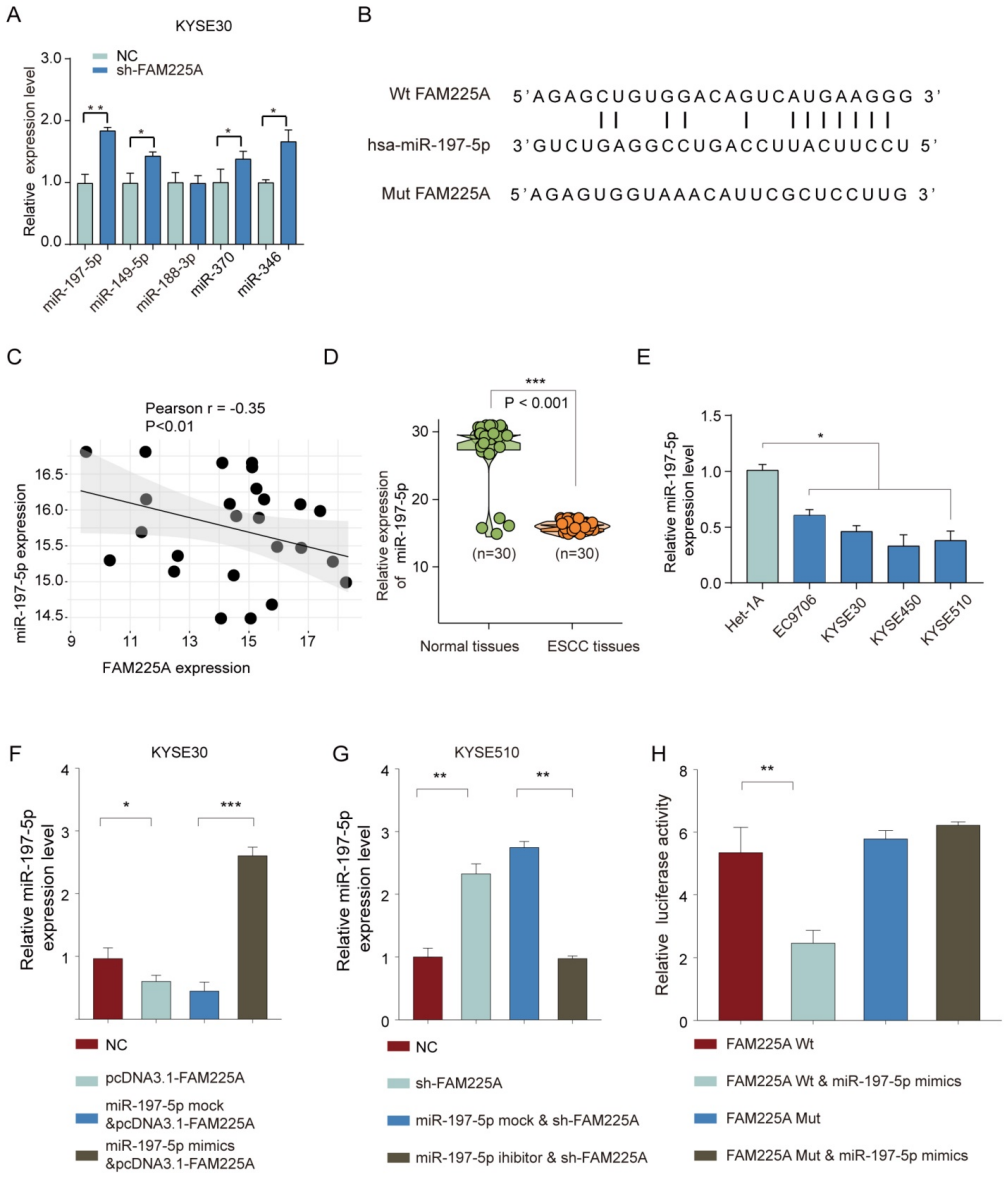
** LncRNA FAM225A functions as a ceRNA via sponging miR-197-5p.** (A) KYSE30 cells were transfected with negative control (NC) or sh-FAM225A. The expression levels of miR-197-5p, miR-149-5p, miR-188-3p, miR-370, and miR-346 were analyzed by qPCR. (B) The putative binding sequences between lncRNA FAM225A and miR-197-5p was shown. (C) Pearson analysis of the correlation between lncRNA FAM225A expression and miR-197-5p expression. (D) The relative expression of miR-197-5p was analyzed in ESCC tissues and non-tumor control tissues by qRT-PCR. (E) The relative expression of miR-197-5p was analyzed in ESCC cell lines and control Het-1A cells by qRT-PCR. (F, G) KYSE30 or KYSE510 cells were transfected with NC, pcDNA3.1-FAM225A, miR-197-5p mock & pcDNA3.1-FAM225A, or miR-197-5p mimics & pcDNA3.1-FAM225A. The relative expression of miR-197-5p was analyzed by qRT-PCR 48 hours post transfection. (H) KYSE510 cells were co-transfected with luciferase reporter vector containing WT or mutated 3'-UTR of lncRNA FAM225A, with or without miR-197-5p mimics. The relative luciferase activity was analyzed 48 hours later by using a dual-Glo luciferase assay kit. * P < 0.05, ** P < 0.01.

To study how lncRNA FAM225A exerts its regulatory function on ESCC, we utilized the online tool StarBase to predict the potential miRNAs interacting with FAM225A. The top five predicted miRNAs with highest score were further evaluated by transfection of sh-FAM225A in KYSE30 cells (**Figure 5A**). LncRNA FAM225A knockdown significantly enhanced miR-197-5p expression and FAM225A had the complementary binding sequences against miR-197-5p (**Figure 5B**). Intriguingly, the miR-197-5p expression was negatively associated with the expression of lncRNA FAM225A in ESCC tissues (**Figure 5C**). Quantitative RT-PCR analysis showed that miR-197-5p expression was markedly lower in ESCC tissues and cell lines than that in non-tumor tissues and control cell line (**Figure 5D and 5E**). In addition, we revealed that overexpression of lncRNA FAM225A suppressed miR-197-5p expression while miR-197-5p mimics reversed the inhibitory effect of lncRNA FAM225A in KYSE30 or KYSE510 cells (**Figure 5F and 5G**). To further validate the relationship between lncRNA FAM225A and miR-197-5p, we performed the luciferase reporter assay and the results revealed that miR-197-5p mimics specifically inhibited the luciferase activity of WT 3'-UTR of lncRNA FAM225A (**Figure 5H**). These results indicated that lncRNA FAM225A sponged miR-197-5p expression in ESCC.

### LncRNA FAM225A exerts its regulatory function on ESCC cell proliferation and metastasis via modulating miR-197-5p expression

**Figure 6:**
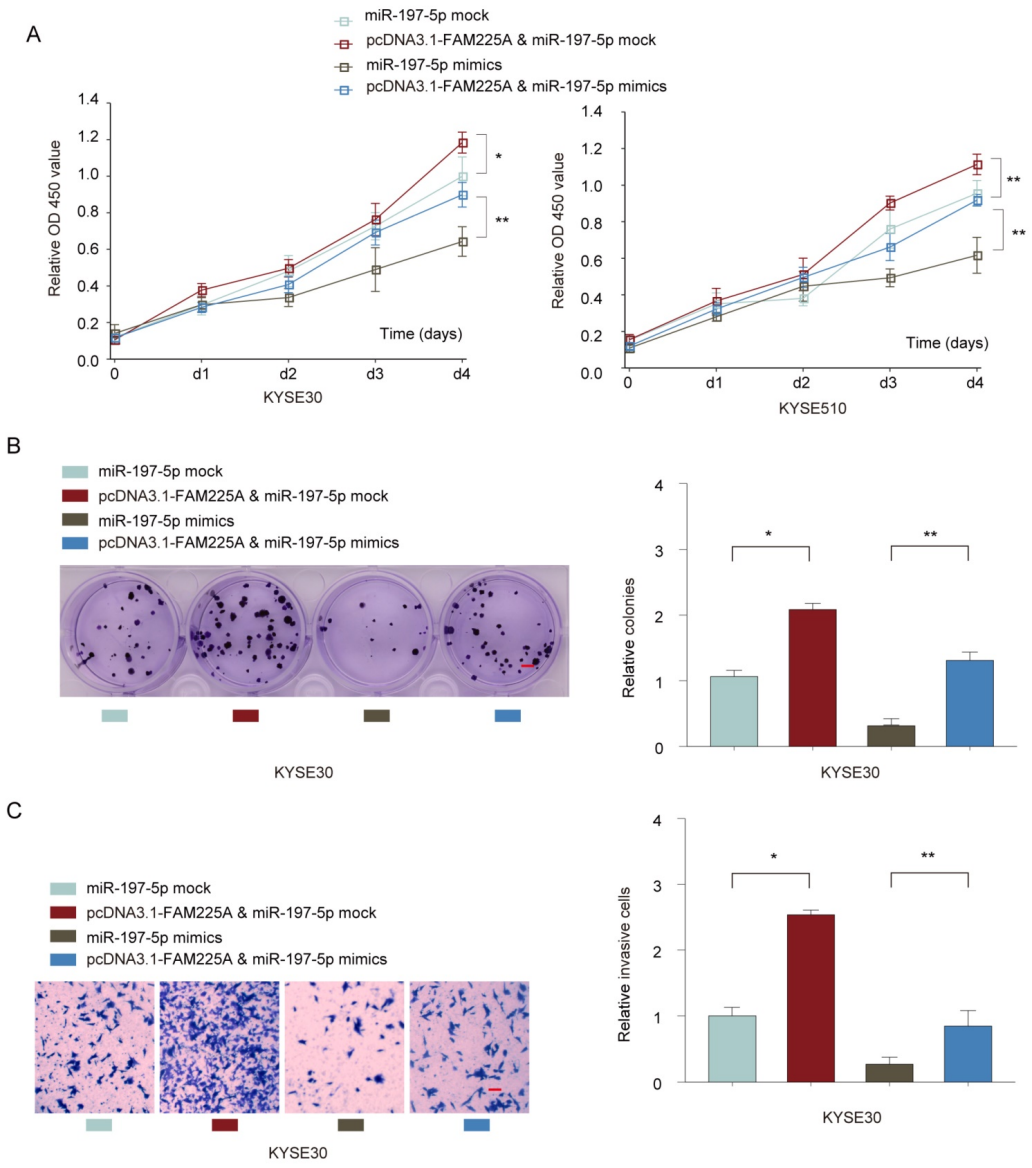
** LncRNA FAM225A exerts its regulatory function on ESCC cell proliferation and metastasis via downregulating miR-197-5p expression.** KYSE30 or KYSE510 cells were transfected with miR-197-5p mock control, pcDNA3.1-FAM225A & miR-197-5p mock, miR-197-5p mimics, or pcDNA3.1-FAM225A & miR-197-5p mimics. (A) Cell proliferation was analyzed by CCK-8 assay. (B) Cell growth was evaluated by colony formation assay. (C) Cell invasion was evaluated by transwell assay. * P < 0.05, ** P < 0.01.

To further study the interaction between lncRNA FAM225A and miR-197-5p, KYSE30 or KYSE510 cells were co-transfected with miR-197-5p mimics or mock control, with or without pcDNA3.1-FAM225A. Cell growth assays demonstrated that overexpression of lncRNA FMA225A enhanced cell growth and miR-197-5p mimics suppressed ESCC cell proliferation (**Figure 6A and 6B**). However, MiR-197-5p overexpression antagonized the promotion effect of pcDNA3.1-FAM225A (**Figure 6A and 6B**). Further, we found that FAM225A promoted ESCC invasion and overexpression of miR-197-5p dampened cell invasion of KYSE30 or KYSE510 cells, whereas overexpression FAM225A together with miR-197-5p showed the similar invasive ability of ESCC cells with control group (**Figure 6C**). Thus, we concluded that lncRNA FAM225A exerted its regulatory effect on ESCC cell proliferation and metastasis via downregulating miR-197-5p expression.

### MiR-197-5p negatively regulates NONO expression and TGF-β signaling in ESCC cells

**Figure 7:**
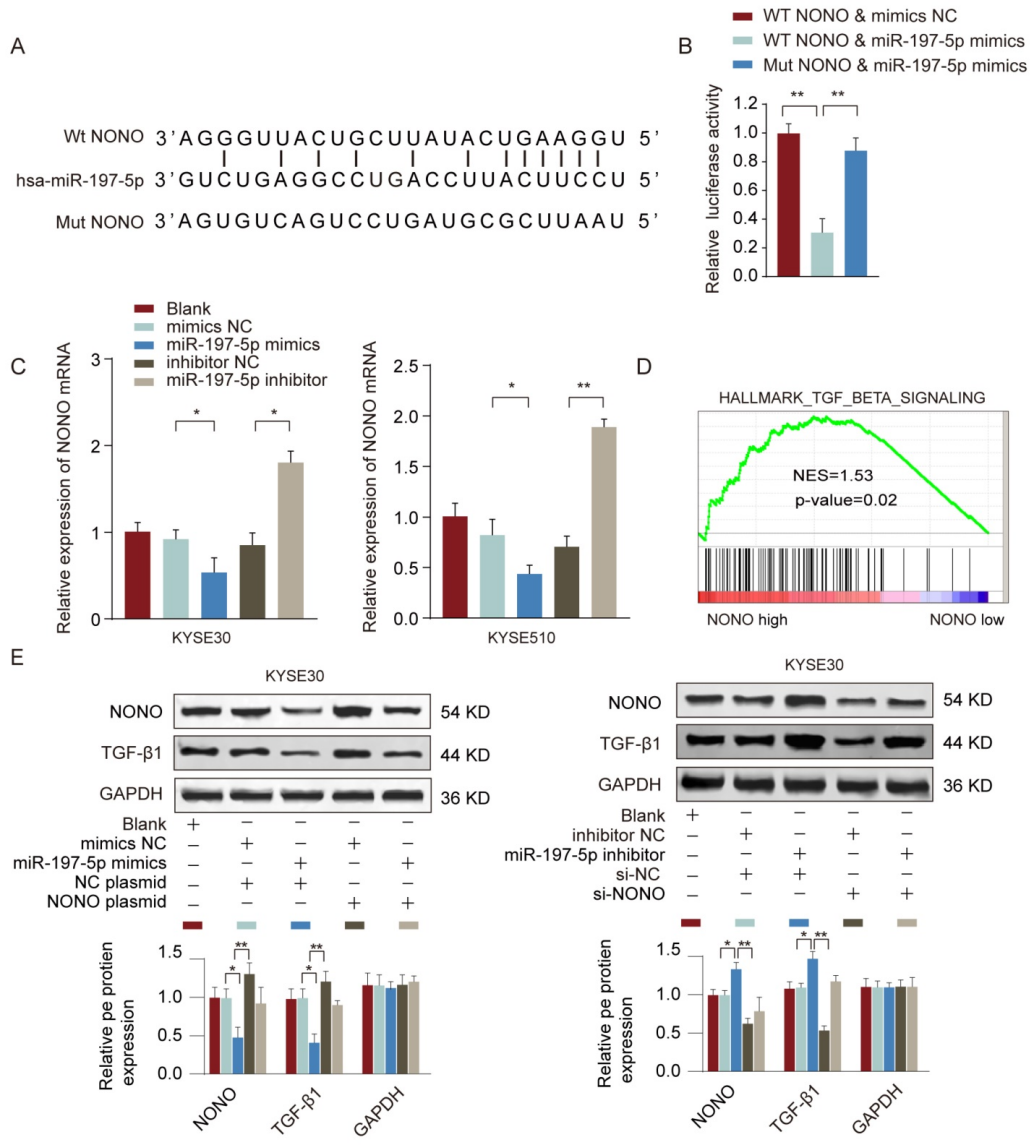
** MiR-197-5p negatively regulates NONO expression and TGF-β signaling in ESCC cells.** (A) The predicted complementary binding sequences between miR-197-5p and NONO was shown. (B) KYSE510 cells were transfected with reporter vector containing WT or mutated 3'-UTR of NONO, together with miR-197-5p mimics or negative control. The relative luciferase activity was analyzed 48 hours post transfection. (C) KYSE30 or KYSE510 cells were transfected with miR-197-5p mimics, miR-197-5p inhibitor or relative negative controls. The relative expression of NONO was analyzed 48 hours later. (D) GSEA analysis revealed TGF-beat signaling signature genes were enriched in NONO high group. (E) KYSE30 or KYSE510 cells were transfected with miR-197-5p mimics or negative control, with or without NONO overexpression plasmid. The protein expression levels of NONO and TGF-β were examined by western blot. * P < 0.05, ** P < 0.01.

To find out the target gene regulated by miR-197-5p, we searched the potential miR-197-5p targets using TargetScan and miRDB online tools. RNA-binding protein NONO was predicted as the top candidate and had the complementary binding sequences of miR-197-5p (**Figure 7A**). Luciferase reporter assay further confirmed the interaction between miR-197-5p and NONO (**Figure 7B**). In addition, we found that miR-197-5p overexpression suppressed NONO expression whereas miR-197-5p inhibition enhanced NONO expression in ESCC cells (**Figure 7C**). We also performed Gene Set enrichment Analysis (GSEA) to investigate the potential signaling pathways involved in miR-197-5p/NONO regulation of ESCC. As shown in **Figure 7D**, TGF-β signaling signature genes were enriched in NONO high expression group. Moreover, we confirmed that in KYSE30 or KYSE510 cells, overexpression of miR-197-5p inhibited MONO and TGF-β expression and overexpression of MONO restored MONO and TGF-β levels in ESCC cells transfected with miR-197-5p (**Figure 7E**). These data suggested that miR-197-5p negatively regulated NONO expression and TGF-β signaling in ESCC cells.

## Discussion

LncRNAs have been demonstrated to play crucial roles in tumorigenesis and metastasis of ESCC 8. LncRNAs could be utilized as early diagnostic biomarkers or prognosis predictors 20, 21. They exert regulatory function via binding to miRNA expression or transcription factors as decoys or acting as dynamic scaffold to transiently assemble with cofactors 22. In this study, we identified lncRNA FAM225A functioned as a ceRNA via sponging miR-197-5p and modulating the expression of NONO to promote the ESCC development and progression. Knockdown of FAM225A inhibited ESCC proliferation and metastasis. These findings demonstrate that FAM225A is an oncogenic lncRNA in ESCC, which could be utilized as therapeutic target for ESCC treatment.

LncRNA FAM225A was identified via microarray screening to be one of the most upregulated lncRNA in nasopharyngeal carcinogenesis. FAM225A enhanced ITGB3 expression via sponging miR-590-3p and miR-1275 17. In another study, FAM225A was overexpressed in colorectal cancer tissues and cell lines and predicted unfavorable outcome of colorectal cancer patients 18. Similar to the published results, we also demonstrated that lncRNA FAM225A was highly expressed in ESCC and functioned as an oncogenic lncRNA. However, we validated that in ESCC, FAM225A interacted with miR-197-5p and the expression of FAM225A was significantly negative associated with miR-197-5p expression in ESCC tissues.

MiR-197-5p was demonstrated to act as circulating biomarker for heart failure 23. It has been reported that in carcinogenesis of fibrosarcoma, miR-197-5p exerts its regulatory role through inhibiting of KIAA0101 24. MiR-197-5p Overexpression markedly suppressed fibrosarcoma cell proliferation and promoted cell apoptosis 24. In glioma, miR-197-5p interacted with lncRNA DLX6-AS1 and suppressed transcription factor E2F1 expression, thus dampened glioma development and progression 25. We revealed the function of miR-197-5p as a tumor suppressor in ESCC. MiR-197-5p expression was downregulated in ESCC tissues and cell lines. Overexpression of miR-197-5p antagonized the oncogenic function of FAM225A in ESCC cells.

The RNA binding protein NONO has been demonstrated to participate in various biological processes including tumorigenesis and metastasis 26. Upregulation of NONO expression is correlated with unfavorable prognosis with multiple tumors, such as bladder cancers, prostate cancers, and malignant melanoma 27-29. Rui Cheng et al. showed that NONO expression was dramatically upregulated in ESCC and knockdown of NONO using siRNA inhibited ESCC cell proliferation and promoted ESCC apoptosis 30. However, in that study, how NONO expression was regulated was not clear. Consistently, we found that NONO was upregulated in ESCC and it was a direct target of miR-197-5p. Moreover, we demonstrated that miR-197-5p negatively regulated NONO expression and TGF-β1 signaling. Nevertheless, the contribution of TGF-β1 signaling to ESCC development and progression needs further investigation.

In conclusion, we explored the expression and function effects of lncRNA FAM225A, as well as the underlying regulatory mechanisms in ESCC development and progression. Our findings indicate that lncRNA FAM225A promotes ESCC tumorigenesis and metastasis via sponging miR-197-5p and upregulating NONO expression. The regulatory axis of FAM225A/miR-197-5p/NONO might be utilized to develop novel therapy for ESCC patients.

## Abbreviations

ESCC: Esophageal squamous cell carcinoma
TMA: Tissue microarray
RT-qPCR: Real-time reverse transcription polymerase chain reaction
IHC: Immunohistochemical
ISH: In Situ Hybridization
OS: Overall survival
DFS: Disease-free survival
TCGA: Cancer Genome Atlas
